# Targeting Myeloperoxidase to Reduce Neuroinflammation in X‐Linked Dystonia Parkinsonism

**DOI:** 10.1111/cns.70109

**Published:** 2024-11-05

**Authors:** Tiziana Petrozziello, Negin Jalali Motlagh, Ranee Zara B. Monsanto, Dan Lei, Micaela G. Murcar, Ellen B. Penney, D. Cristopher Bragg, Cara Fernandez‐Cerado, G. Paul Legarda, Michelle Sy, Edwin Muñoz, Mark C. Ang, Cid Czarina E. Diesta, Can Zhang, Rudolph E. Tanzi, Irfan A. Qureshi, John W. Chen, Ghazaleh Sadri‐Vakili

**Affiliations:** ^1^ Sean M. Healey & AMG Center for ALS at Mass General Massachusetts General Hospital Boston Massachusetts USA; ^2^ Department of Radiology, Institute for Innovation in Imaging Massachusetts General Hospital Boston Massachusetts USA; ^3^ Center for Systems Biology Massachusetts General Hospital and Harvard Medical School Boston Massachusetts USA; ^4^ Department of Neurology Genetics and Aging Research Unit, McCance Center for Brain Health, MassGeneral Institute for Neurodegenerative Disease Massachusetts General Hospital Boston Massachusetts USA; ^5^ Department of Neurology Massachusetts General Hospital Boston Massachusetts USA; ^6^ Sunshine Care Foundation Roxas City Capiz Philippines; ^7^ Department of Pathology College of Medicine, University of the Philippines Manila Philippines; ^8^ Department of Neurosciences Makati Medical Center Makati Philippines; ^9^ Biohaven Pharmaceuticals Inc. New Haven Connecticut USA

**Keywords:** inflammation, myeloperoxidase, verdiperstat, X‐linked dystonia parkinsonism

## Abstract

**Aims:**

Although the genetic locus of X‐linked dystonia parkinsonism (XDP), a neurodegenerative disease endemic in the Philippines, is well‐characterized, the exact mechanisms leading to neuronal loss are not yet fully understood. Recently, we demonstrated an increase in myeloperoxidase (MPO) levels in XDP postmortem prefrontal cortex (PFC), suggesting a role for inflammation in XDP pathogenesis. Therefore, we hypothesized that inhibiting MPO could provide a therapeutic strategy for XDP.

**Methods:**

MPO activity was measured by using an MPO‐activatable fluorescent agent (MAFA) in human postmortem PFC. Reactive oxygen species (ROS) and MPO activity were measured in XDP‐derived fibroblasts and SH‐SY5Y cells following MPO inhibition.

**Results:**

MPO activity was significantly increased in XDP PFC. Additionally, treatment of cell lines with postmortem XDP PFC resulted in a significant increase in ROS levels. To determine whether increases in MPO activity caused increases in ROS, MPO content was immunodepleted from XDP PFC, which resulted in a significant decrease in ROS in SH‐SY5Y cells. Consistently, the treatment with verdiperstat, a potent and selective MPO inhibitor, significantly decreased ROS in both XDP‐derived fibroblasts and XDP PFC‐treated SH‐SY5Y cells.

**Conclusions:**

Collectively, our results suggest that MPO inhibition mitigates oxidative stress and may provide a novel therapeutic strategy for XDP treatment.

## Graphical abstract

Myeloperoxidase activity is increased in X‐linked dystonia parkinsonism (XDP) postmortem brain and caused an increase in oxidative stress. Decreasing MPO levels or inhibiting its activity significantly reduced reactive oxygen species in vitro, suggesting that targeting MPO may reduce oxidative stress in XDP.

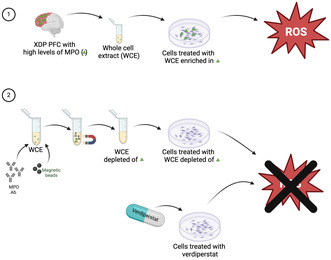

## 1 Introduction

X‐linked dystonia parkinsonism (XDP) is an adult‐onset, progressive, debilitating movement disorder that predominantly affects males with maternal ancestry originating from the island of Panay in the Philippines [1]. All affected individuals reported to date share a common disease haplotype consisting of multiple sequence variants in and around the *TAF1* gene, which encodes for the TATA‐binding protein‐associated factor‐1 (TAF1) [2, 3, 4, 5, 6]. All of these variants fall within non‐coding gene regions, and only one has been directly linked to disease pathogenesis: a SINE‐VNTR‐Alu (SVA)‐type retrotransposon insertion within intron 32 of *TAF1* [3, 5]. This XDP‐specific SVA element includes a 5′ tandem repeat, (CCCTCT)_n_, the length of which inversely correlates with the age of disease onset in patients [6, 7], and this insertion disrupts transcription of *TAF1* in XDP cell models [5, 8]. However, the exact molecular mechanisms by which these perturbations in *TAF1* lead to neuronal loss in XDP are still not completely elucidated.

Similar to other neurodegenerative diseases, such as Alzheimer's disease (AD) [9], Huntington's disease (HD) [10], Parkinson's disease (PD) [11], and amyotrophic lateral sclerosis (ALS) [12], a role for neuroinflammation in XDP pathogenesis has recently been suggested. Specifically, alterations in inflammatory pathways, including the dysregulation of NFκB signaling, hypersensitivity to TNF‐α, and increases in several proinflammatory markers, were described in XDP‐derived fibroblasts and neuronal stem cells (NSCs) [13]. Additionally, we have demonstrated an increase in astrogliosis and microgliosis as well as myeloperoxidase (MPO) levels in XDP postmortem prefrontal cortex (PFC), further supporting a role for neuroinflammation in XDP pathogenesis [14].

MPO is a member of the heme peroxidase family released from peripheral immune cells, such as neutrophils, macrophages, and monocytes [15], as well as from reactive astrocytes and activated microglia [16, 17, 18, 19, 20, 21]. Of note, prolonged increases in systemic levels of MPO have been linked to tissue damage during chronic inflammation, mostly due to degranulation and apoptosis of immune cells as well as prolonged increases in reactive oxygen species (ROS) [22, 23, 24, 25]. Therefore, we sought to determine whether increases in MPO contribute to neuroinflammation in XDP pathogenesis. First, we measured MPO activity in a large cohort of human postmortem XDP PFC by using a novel MPO‐activatable fluorescent molecular imaging probe (MAFA) [26]. Next, we assessed the downstream consequences of increases in MPO in cellular models of XDP. Lastly, we sought to determine whether inhibiting MPO mitigates inflammation in XDP in vitro.

## 2 Materials and Methods

### 2.1 Human Brain Tissue Samples

Postmortem XDP PFC were provided by the Massachusetts General Hospital Collaborative Center for XDP (CCXDP) with approval of the Institutional Review Boards (IRB) at Massachusetts General Hospital (Boston, USA) and Makati Medical Center (Makati City, Philippines). Detailed methods on donor consent, tissue collection, and processing, as well as quality control metrics, have been previously reported [27]. Postmortem control PFC were provided by the Massachusetts Alzheimer's Disease Research Center (ADRC) with approval from the Massachusetts General Hospital IRB. This study included only control and XDP PFC derived from male subjects. Demographic information about the samples is provided in Table 1.

**TABLE 1:** Postmortem prefrontal cortex (BA9) sample information.

	PMI (h)	Age at disease onset	Age at death	Disease duration (months)	Repeat size within SVA
Control 1	Unknown	Not applicable	85	Not applicable	Not applicable
Control 2	36	Not applicable	88	Not applicable	Not applicable
Control 3	16	Not applicable	63	Not applicable	Not applicable
Control 4	23	Not applicable	> 90	Not applicable	Not applicable
Control 5	36	Not applicable	> 90	Not applicable	Not applicable
Control 6	24	Not applicable	77	Not applicable	Not applicable
Control 7	Unknown	Not applicable	59	Not applicable	Not applicable
XDP 1	6.36	46	51	60	36
XDP 2	29	34	41	84	41
XDP 3	19.5	42	49	84	46
XDP 4	17	40	46	72	42
XDP 5	35.52	44	49	60	36
XDP 6	23.75	27	43	192	42
XDP 7	7.8	32	41	108	42
XDP 8	22.21	32	49	204	42
XDP 9	10.28	26	36	120	52
XDP 10	14.62	49	55	72	39
XDP 11	9.75	47	59	144	45
XDP 12	35.32	30	46	192	44
XDP 13	13.98	45	46	12	34
XDP 14	24	33	42	108	42
XDP 15	25.08	49	59	120	54
XDP 16	11.16	39	47	96	40
XDP 17	13.3	41	56	180	41
XDP 18	7.8	38	40	24	41
XDP 19	10.67	30	32	24	50
XDP 20	7.67	49	60	132	36
XDP 21	4.88	51	58	84	38
XDP 22	10.95	51	63	144	36
XDP 23	Unknown	50	54	48	41
XDP 24	18.05	59	65	72	41
XDP 25	9.15	33	40	84	45

Although XDP is mainly characterized by loss of medium spiny neurons in the striatum, previous studies have provided evidence demonstrating that other brain areas such as frontal and temporal cortices, pallidum, and cerebellum also degenerate in XDP [28, 29, 30, 31, 32]. Our previous findings support these studies and demonstrated a significant increase in neuroinflammation and MPO in XDP PFC [33]. Therefore, we have continued our work assessing and understanding the precise molecular mechanisms that occur in the PFC in XDP.

### 2.2 Ex Vivo Detection of MPO Activity Using MAFA

As a substrate for MPO, MPO can oxidize MAFA to form radicals that bind to nearby proteins containing phenolic or indolic moieties. Activated MAFA would then remain bound to tissues after washing, while inactivated agents would be washed away, enabling the identification of areas with elevated MPO activity [26]. Fresh‐frozen human brain sections (8 μm thickness) were fixed in 4% paraformaldehyde (PFA) for 5 min at room temperature (RT) and then incubated in blocking solution containing 1% fetal bovine serum (FBS; #A5670701, Gibco, Thermo Fisher Scientific, MA), and 0.3% Triton X‐100 (#T8787‐50 mL, MilliporeSigma, MA) at RT for 1 h. Following three washes in phosphate buffer saline (PBS; #10010049, Gibco, Thermo Fisher Scientific, MA), the sections were incubated with MAFA (1:300 of stock solution 10 mM dimethyl‐sulfoxide DMSO; #D1391, Thermo Fisher Scientific, MA) and 1 mM of 3% hydrogen peroxide (H_2_O_2_; #BP2633500, Thermo Fisher Scientific, MA) for 30 min at RT. Lastly, the sections were mounted in an antifade mounting medium (#ZJ0808, Vectashield, Vector Laboratories, CA) containing the nuclear stain DAPI. Images were captured with a Nikon DS‐Ri2 model microscope connect to Prime BSI Express. Intensity of fluorescence was measured by using ImageJ 1.53 t (National Institute of Health, Bethesda, MD).

### 2.3 Meso Scale Discovery (MSD) Assay

Cytokines levels were assessed using the human proinflammatory panel‐1 10‐plex kits to detect 10 cytokines, including interferon γ (INF‐γ), interleukin (IL)‐1β, IL‐2, IL‐4, IL‐5, IL‐6, IL‐8, IL‐10, IL‐12p70, IL‐13, and tumor necrosis factor‐α (TNF‐α). Specifically, cytokines levels were measured using an electrochemiluminescence‐based multiarray method through the Quickplex SQ 120 system (Meso Scale Diagnostics LLC, MD) following previously reported methods [34, 35]. Briefly, both experimental samples and protein standards provided by the manufacturer were incubated on a shaker at 4°C overnight. Next, experimental samples and protein standards were washed off the plates and then incubated with the detection antibodies provided by the kit at room temperature while shaking for 2 h, followed by washing and the addition of the reading buffer. Lastly, the electrochemiluminescence signals were captured by the SQ 120 system, and cytokines concentrations (pg/mL) were calculated following manufacturer's instruction using the standard concentrations provided by the kit.

### 2.4 Cell Cultures

#### 2.4.1 Human Fibroblast Cultures

Human fibroblasts were provided by the CCXDP at Massachusetts General Hospital. In this study, we used two fibroblast lines derived from individuals who lived with XDP and their unaffected family members that were previously described [5, 13, 36]. All fibroblasts were grown in Dulbecco's Modified medium (DMEM; #11965118, Gibco, Thermo Fisher Scientific, MA) supplemented with 20% FBS (#A5670701, Gibco, Thermo Fisher Scientific, MA), and 1x penicillin/streptomycin/L‐glutamine (#10378016, Thermo Fisher Scientific, MA) and kept in an incubator at 37°C, 5% CO_2_. Specifically, this study was performed in fibroblasts derived from two individuals living with XDP (33109 and 35883) and two controls (36175 and 33362).

#### 2.4.2 SH‐SY5Y Cells

Human neuroblastoma SH‐SY5Y cells (ATCC CRL‐2266) were cultured in DMEM/Nutrient Mixture F‐12 (1:1) supplemented with 10% inactivated FBS (#A5670701, Gibco, Thermo Fisher Scientific, MA), 2 mM L‐glutamine, 50 μg/mL streptomycin, and 50 IU/mL penicillin (#A5670701, Gibco, Thermo Fisher Scientific, MA). The cells were kept at 5% CO_2_ at 37°C.

### 2.5 Oxidative Stress Detection

ROS levels were measured in both fibroblasts and SH‐SY5Y cells by using CellROX Orange reagent (#C10443; Thermo Fisher Scientific, MA), a fluorogenic probe for measuring oxidative stress in live cells, as previously reported [37]. Briefly, following specific treatments, the cells were incubated with 2.5 μM CellROX for 30 min at 37°C in the dark. Then, the medium was replaced with fresh culture media before imaging on a BioTek Cytation 5 imaging reader (BioTek, VT). Images were captured from 6 random areas of each well every 10 min for 2 h. To analyze data generated by Cytation, images were processed using a custom Fiji macro to automatically calculate mean fluorescent intensity (MFI) for each image. The MFI values were then grouped by using a custom Python script, as previously reported [37]. Statistics and XY curves were generated using GraphPad Prism 10.2.0.

### 2.6 MPO Activity Assay

MPO activity was measured by using the OxiSelect Myeloperoxidase Chlorination Activity Assay Kit (#STA‐803; Cell Biolabs, CA), according to manufacturer's protocol. Briefly, samples were incubated in 1 mM hydrogen peroxide (H_2_O_2_) solution for 1 h at RT. Next, samples were incubated in 1x stop solution for 15 min at RT before incubating in 1 mM chromogen working solution for additional 15 min in the dark at RT. All solutions were provided by the kit. At the end of the last incubation, a standard curve was generated following manufacturer's instruction, and absorbance was read at 405 nm. MPO activity in milliunits/mL (mU/mL) was determined for each sample by dividing the quantity of chromogen consumed by the reaction time as indicated by the kit's instruction.

### 2.7 MPO Immunodepletion

MPO content was immunodepleted from four XDP PFC homogenates that contained the highest MPO levels, as determined by previously published studies [14]. Specifically, 150 μg of proteins from each XDP PFC was incubated with 15 μL anti‐MPO antibody (#A1374, ABclonal Technology, MA) in GAL4 immunoprecipitation buffer, composed of 250 mM sodium chloride (NaCl; #S9888, Sigma‐Aldrich, MA), 5 mM ethylenediaminetetraacetic acid (EDTA; #AM9260G, Thermo Fisher Scientific, MA), 1% Nonidet P‐40 (#J19628.K2, Thermo Fisher Scientific, MA), 50 mM Tris, pH7.5 (#AM9850G, Thermo Fisher Scientific, MA). After the incubation at 4°C for 3.5 h, magnetic protein A beads (Invitrogen, Thermo Fisher, MA) were incubated (20 μL per sample) in agitation overnight at 4°C. At the end of the incubation, all samples were placed on a magnetic rack to separate the supernatants, depleted of MPO. The supernatants were then collected and saved in new tubes labeled as XDP(−). The magnetic beads bound to MPO were, instead, resuspended in GAL4 buffer (50 μL per sample), boiled at 95°C for 5 min and magnetized again to remove the magnetic beads and save the supernatants, enriched in MPO, in new tubes labeled as XDP(+). The efficiency of the immunodepletion was determined by measuring MPO activity in whole cell extract from XDP, XDP(−), and XDP(+) PFC.

### 2.8 Cell Cultures Treatment

#### 2.8.1 XDP PFC Treatment

SH‐SY5Y cells were incubated with 10 ng/mL of homogenates from either XDP PFC (*n* = 4), XDP(−) (*n* = 4) or XDP(+) (*n* = 4) for 24 h before measuring MPO activity and ROS as described above.

#### 2.8.2 Verdiperstat

Control‐ and XDP‐derived fibroblasts were treated with either 0.1, 0.5, 1, 5, or 10 μg/mL of verdiperstat for 24 h before measuring MPO activity. ROS levels were measured in control‐ and XDP‐derived fibroblasts in the absence or in the presence of 5 μg/mL of verdiperstat. Similarly, MPO activity and ROS were assessed in XDP‐treated SH‐SY5Y cells in the absence or in the presence of 5 μg/mL of verdiperstat.

### 2.9 Statistics

Normal distribution of data was not assumed regardless of sample size or variance. Individual value plots with the central line representing the median and the whiskers representing the interquartile range, box plot with the central line representing the median, the edges representing the interquartile range, and the whiskers representing the minimum and maximum values, and XY curves were used for graphical representation. Comparisons between groups were performed using a non‐parametric Mann–Whitney *U* test, a one‐way ANOVA followed by Tukey's test, and a two‐way ANOVA followed by Tukey's test. Correlation of MPO levels with XDP clinical features, including age at disease onset, age at death, disease duration, and repeat size within the SVA, were performed as non‐parametric Spearman correlations. All tests were two‐sided with a significance level of 0.05, and exact *p* values were reported. GraphPad Prism 10.2.0 was used to perform statistical analyses and generate graphs.

### 2.10 Study Approval

The study was approved by the Partners Healthcare Institutional Review Board at Massachusetts General Hospital (Boston, MA, USA) and by the Makati Medical Center (Makati City, Philippines). Postmortem consent was obtained from the appropriate representative (next of kin or health care proxy) prior to autopsy.

## 3 Results

### 3.1 MPO Activity Was Increased in XDP Postmortem PFC

**FIGURE 1:**
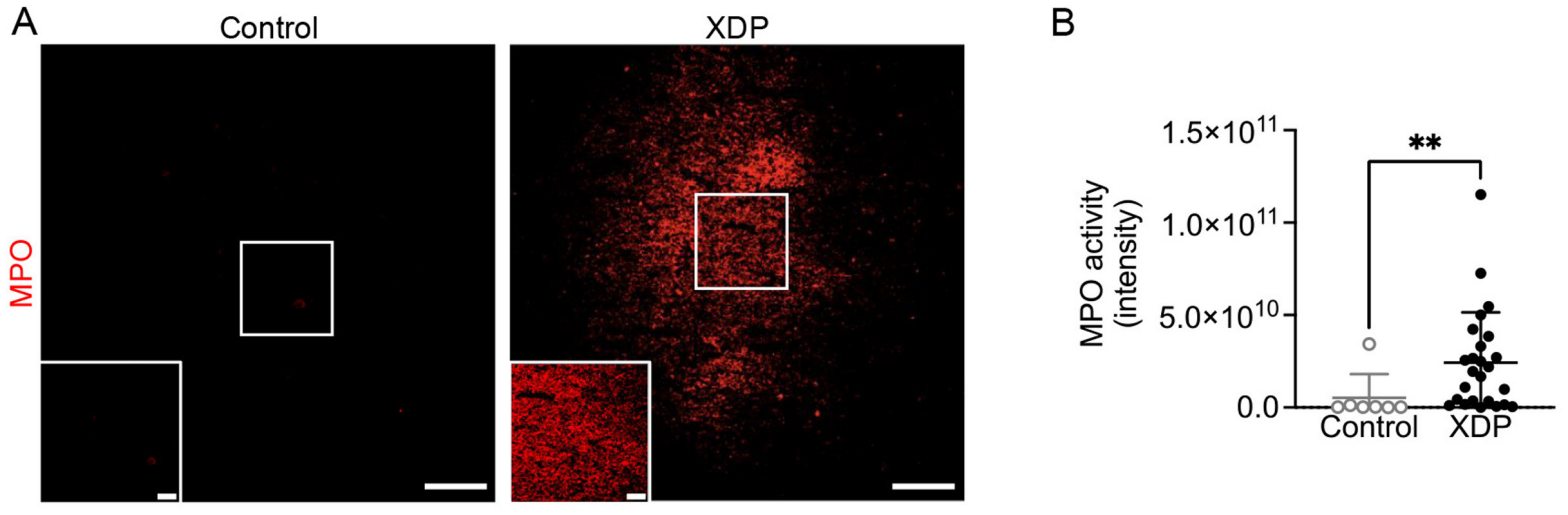
MPO activity was increased in human postmortem XDP PFC. (A) Representative images of MPO activity in control and XDP postmortem PFC. (B) MPO activity was significantly increased in XDP PFC (*n* = 25) compared with controls (*n* = 7) (Mann–Whitney *U* test = 28, *p* = 0.0051). **p < 0. 01. Scale bar: 100 μm; scale bar insert: 25 μm.

Given the previously reported increase in MPO levels in XDP PFC [14], we measured MPO activity in brain tissue sections ex vivo using a specific MPO activity fluorescent imaging probe (MAFA) in a large cohort of postmortem XDP PFC [26]. Our results revealed a significant increase in MPO activity in XDP PFC compared with controls (Figure 1), consistent with our previously published findings.

**FIGURE 2:**
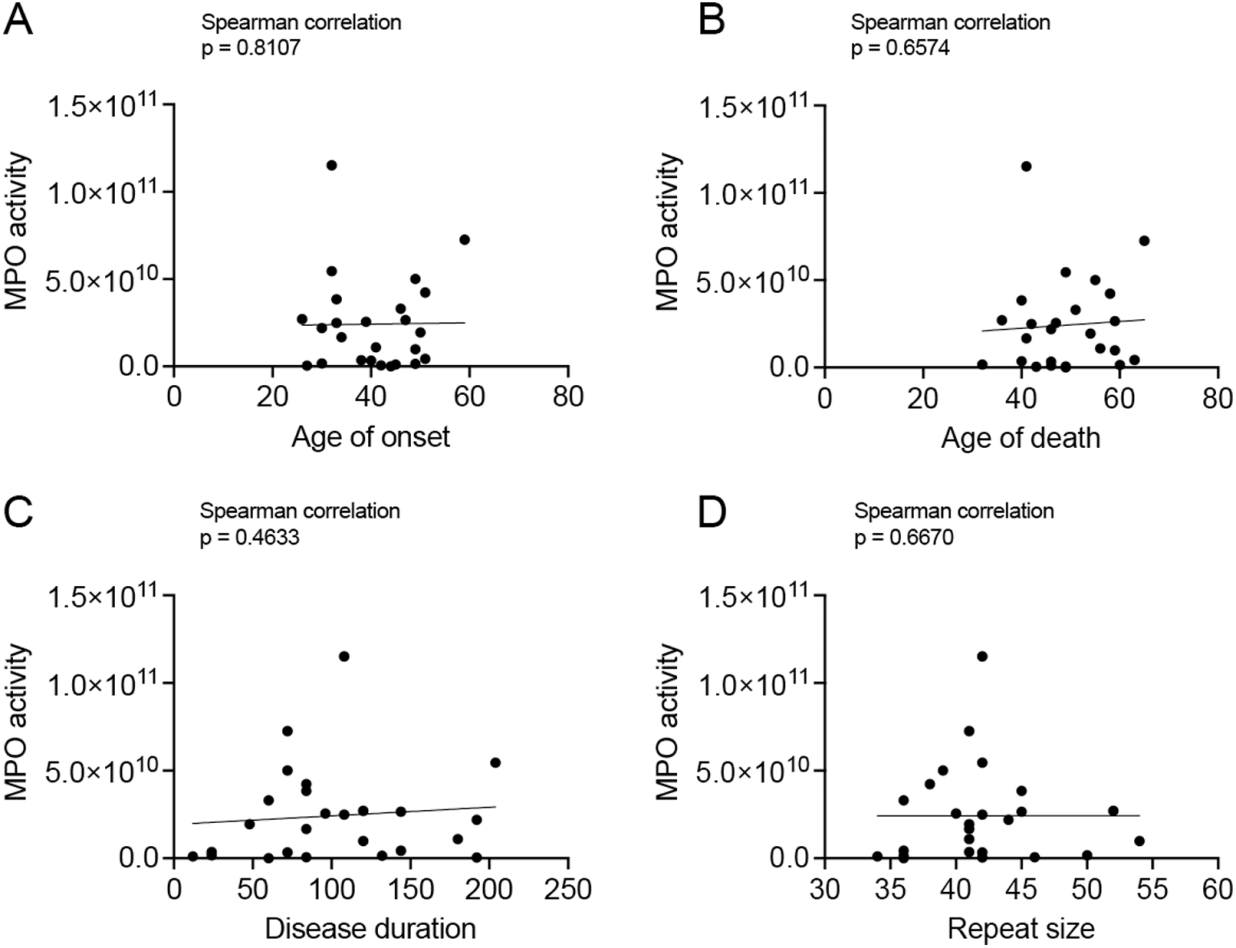
There were no correlations between MPO activity and XDP clinical parameters. There was no impact of (A) age at disease onset (Spearman correlation, *r* = 0.05046, *p* = 0.8107), (B) age at death (Spearman correlation, *r* = 0.09327, *p* = 0.6574), (C) disease duration (in months) (Spearman correlation, *r* = 0.1537, *p* = 0.4633), and (D) repeat size within the SVA (Spearman correlation, *r* = 0.09051, *p* = 0.6670) on MPO activity in XDP PFC (*n* = 25).

To determine whether there was an association between known XDP clinical parameters and MPO activity, we correlated MPO activity in PFC with age at disease onset, age at death, disease duration, and the size of the hexameric repeat expansion within the SVA in *TAF1* for all XDP samples. The results demonstrated no impact or correlation of any of the clinical parameters with MPO activity in XDP (Figure 2).

### 3.2 Cytokines Levels Were Not Changed in XDP Postmortem PFC

Given that increases in MPO result in increases in proinflammatory cytokines [21, 38, 39], we measured alterations in a panel of 10 cytokines in control and XDP postmortem PFC using an MSD assay. Our results demonstrated that the levels of INF‐γ, TNF‐α, IL‐1β, IL‐2, IL‐4, IL‐6, IL‐8, IL‐10, IL‐12p70, and IL‐13 were not significantly different between XDP and control PFC (Figure S1).

### 3.3 ROS Levels were Increased in XDP

**FIGURE 3:**
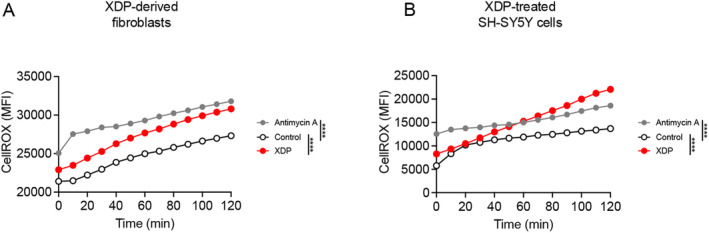
ROS levels were increased in XDP‐derived fibroblasts and XDP‐treated SH‐SY5Y cells. (A) Two‐way ANOVA demonstrated a significant effect of genotype ([*F* (2, 24) = 244.5], *p* < 0.0001), and time ([*F* (12, 24) = 47.84], *p* < 0.0001) on ROS levels in XDP fibroblasts. As revealed by Tukey's test, ROS levels were increased in antimycin A‐treated fibroblasts compared with vehicle‐treated control fibroblasts (*p* < 0.0001). Additionally, there was a significant increase in ROS in vehicle‐treated XDP fibroblasts compared with control fibroblasts (*p* < 0.0001). XY graph represents the average ROS measured in fibroblasts derived from two individuals living with XDP (33,109 and 35,883) and two controls (36,175 and 33,362). (B) Two‐way ANOVA demonstrated a significant effect of treatment ([*F* (2, 24) = 25.66], *p* < 0.0001), and time ([*F* (12, 24) = 8.994], *p* < 0.0001) on ROS levels in SH‐SY5Y cells. As expected, Tukey's test revealed a significant increase in ROS in antimycin A‐treated cells compared with vehicle‐treated cells (*p* < 0.0001). Importantly, there was a significant increase in ROS in XDP‐treated cells compared with vehicle‐treated cells (*p* < 0.0001). ****p < 0.0001.

Increases in MPO activity are also directly linked to increases in ROS by generating hypochlorous acid [22, 23]. Therefore, we measured ROS in XDP‐derived fibroblasts by using CellROX and live cell imaging. Antimycin A, an inhibitor of mitochondrial complex III capable of increasing ROS [40], was used as positive control. As expected, ROS levels were significantly increased in antimycin A‐treated cells compared with control‐derived fibroblasts. Importantly, our results demonstrated a significant increase in ROS in XDP‐derived fibroblasts compared with control‐derived fibroblasts (Figure 3A).

Next, we sought to determine whether the treatment of SH‐SY5Y cells with whole cell extracts derived from XDP PFC could recapitulate pathological features of XDP, such as increases in oxidative stress, as we have recently demonstrated in ALS [37]. SH‐SY5Y cells were treated with XDP PFC (10 ng/mL) for 24 h before ROS levels were measured as described above. As expected, antimycin A induced a significant increase in ROS compared with vehicle treated cells. Furthermore, there was a significant increase in ROS in XDP treated SH‐SY5Y cells compared with vehicle treated cells (Figure 3B).

### 3.4 MPO Immunodepletion Reduced MPO Activity and ROS In Vitro

**FIGURE 4:**
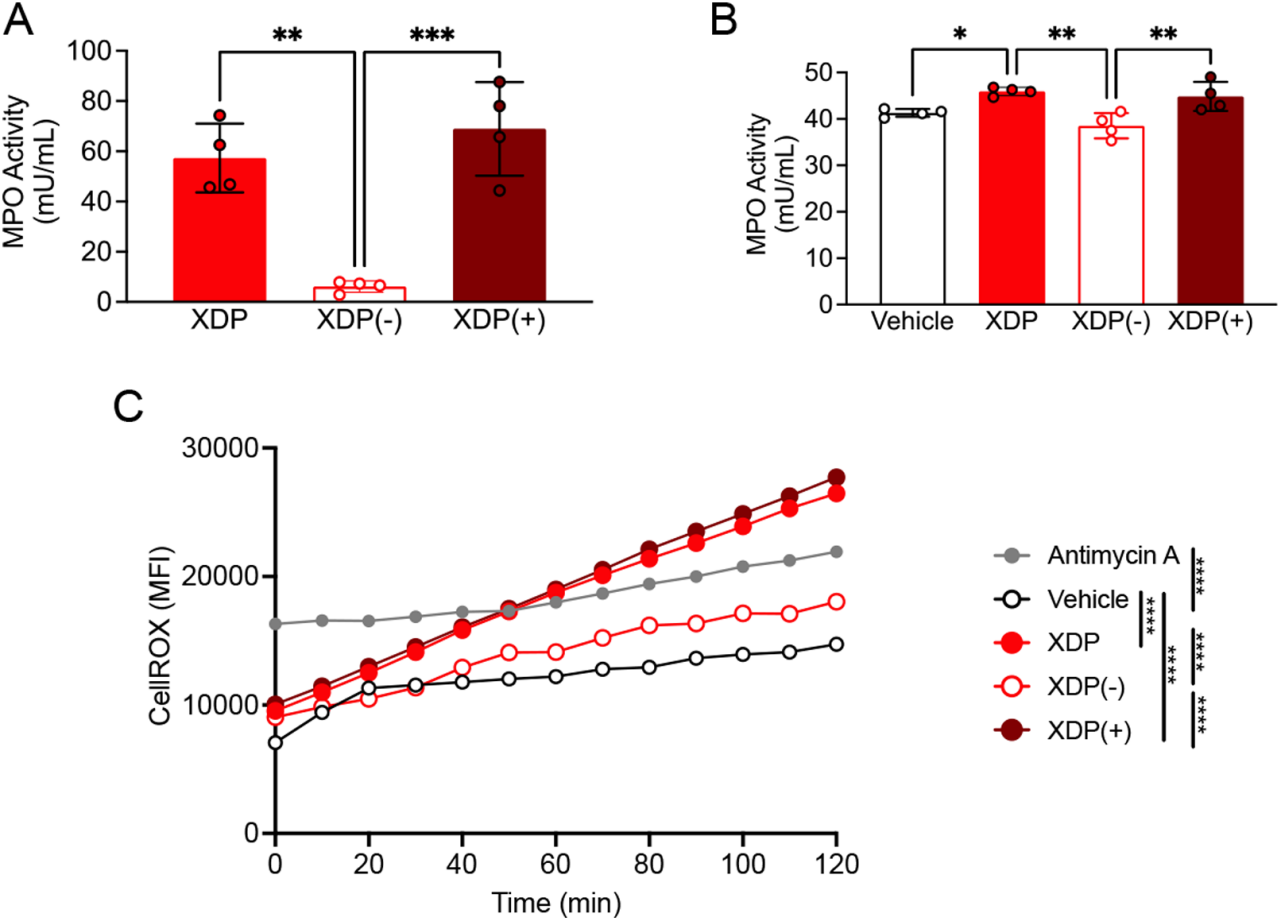
MPO immunodepletion decreased ROS levels in SH‐SY5Y cells. (A) One‐way ANOVA revealed a significant effect of immunodepletion ([*F* (2, 9) = 24.7], *p* = 0.0002) on MPO activity in SH‐SY5Y cells. Specifically, Tukey's test revealed a significant decrease in MPO activity in XDP(−) treated cells compared with both XDP (*p* = 0.011) and XDP(+) treated cells (*p* = 0.0003). (B) One‐way ANOVA demonstrated a significant effect of treatment ([*F* (3, 12) = 9.763], *p* = 0.0015) on MPO activity in SH‐SY5Y cells. Tukey's test demonstrated a significant increase in MPO activity in XDP‐treated cells compared with vehicle‐treated cells (*p* = 0.0460) as well as a significant decrease in MPO activity in XDP(−) cells compared with both XDP (*p* = 0.0020) and XDP(+) treated cells (*p* = 0.0066). (C) Two‐way ANOVA revealed a significant effect of immunodepletion ([*F* (4, 48) = 32.85], *p* < 0.0001) and time ([*F* (12, 48) = 16.96], *p* < 0.0001) on ROS levels in SH‐SY5Y cells. As expected, there was an increase in ROS in antimycin A treated cells compared with vehicle treated cells (Tukey's test, *p* < 0.0001). Additionally, Tukey's test demonstrated a significant increase in ROS in XDP (*p* < 0.0001) and XDP(+) treated cells (*p* < 0.0001) compared with vehicle treated cells. Importantly, Tukey's test also revealed a significant decrease in ROS in XDP(−) treated cells compared with both XDP (*p* < 0.0001) and XDP(+) treated cells (*p* < 0.0001). *p < 0.05; **p < 0.01; ***p < 0.001; ****p < 0.0001.

To determine whether increases in MPO directly induce an increase in ROS in XDP, we removed MPO from XDP postmortem PFC homogenates by performing an immunodepletion experiment. To determine the efficiency of immunodepletion, MPO activity was measured in whole cell extracts from XDP PFC, XDP PFC depleted of MPO [XDP(−)], and XDP PFC enriched in MPO [XDP(+)] by using a commercially available kit. The results revealed that MPO activity was significantly decreased in XDP(−) compared with both XDP(+) and XDP PFC, confirming the successful removal of MPO from the samples (Figure 4A).

Next, SH‐SY5Y cells were treated with XDP, XDP(−), and XDP(+) PFC (10 ng/mL) for 24 h before measuring MPO activity using a commercially available kit. Our findings indicated a significant increase in MPO activity in XDP and XDP(+) treated cells compared with vehicle treated cells. Importantly, MPO activity was significantly decreased in XDP(−) treated cells (Figure 4B).

Lastly, to determine whether MPO causes increase in ROS in XDP, ROS levels were measured in SH‐SY5Y cells following treatment with either XDP, XDP(−), or XDP(+) PFC (10 ng/mL/24 h) by using CellROX and live cell imaging. As expected, the positive control antimycin A induced a significant increase in ROS. A similar increase was measured in XDP and XDP(+) treated cells compared with vehicle treated cells. Importantly, there was a significant decrease in ROS in XDP(−) treated cells compared with both XDP and XDP(+) treated cells (Figure 4C).

### 3.5 Verdiperstat Reduced MPO Activity and ROS in XDP‐Derived Fibroblasts

**FIGURE 5:**
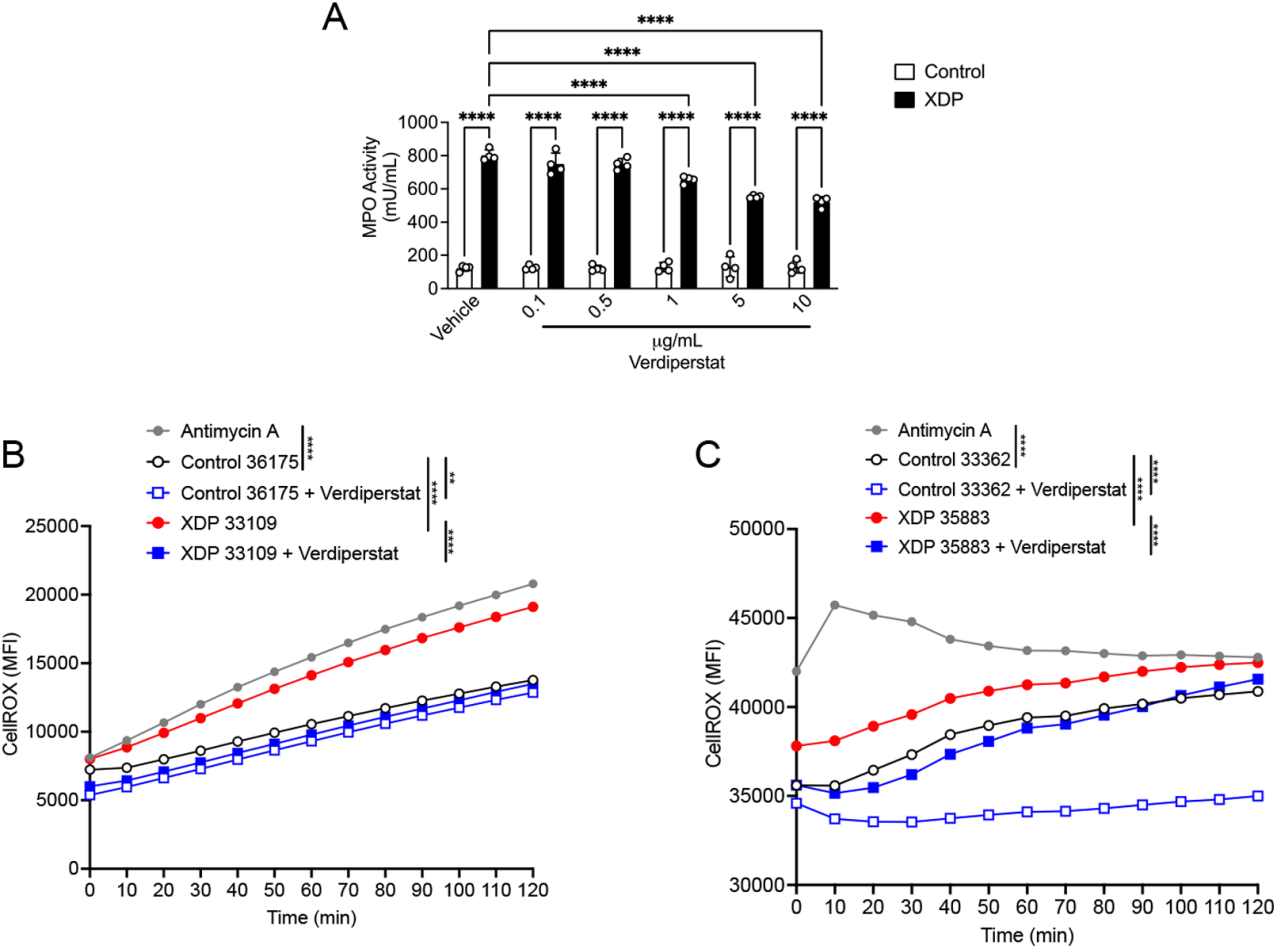
Verdiperstat decreased MPO activity and ROS levels in XDP fibroblasts. (A) Two‐way ANOVA revealed a significant effect of treatment ([*F* (5, 36) = 20.08], *p* < 0.0001), genotype ([*F* (1, 36) = 2890], *p* < 0.0001), and treatment × XDP interaction ([*F* (5, 36) = 22.71], *p* < 0.0001) on MPO activity in fibroblasts. Tukey's' test demonstrated a significant increase in MPO activity in XDP‐ compared with control‐derived fibroblasts (*p* < 0.0001) as well as a significant increase in MPO activity in XDP‐derived fibroblasts treated with either 0.1 (*p* < 0.0001), 0.5 (*p* < 0.0001), 1 (*p* < 0.0001), 5 (*p* < 0.0001), or 10 μg/mL of verdiperstat (*p* < 0.0001) compared with control‐derived fibroblasts treated with the same doses of verdiperstat. Lastly, Tukey's test revealed a significant decrease in MPO activity in XDP‐derived fibroblasts treated with either 5 (*p* < 0.0001) or 10 μg/mL of verdiperstat (*p* < 0.0001) compared with vehicle‐treated XDP‐derived fibroblasts. Bar graph represents the average MPO activity measured in two different XDP fibroblast lines (33109 and 35883) and two different control fibroblasts (36175 and 33362). (B) Two‐way ANOVA revealed a significant effect of genotype ([*F* (4, 48) = 120.3], *p* < 0.001), and time ([*F* (12, 48) = 61.32], *p* < 0.0001) on ROS levels in fibroblasts. Tukey's test revealed an expected significant increase in ROS in antimycin A‐treated fibroblasts compared to vehicle‐treated control fibroblasts (*p* < 0.0001). There was also a significant with in ROS in XDP fibroblasts compared to control fibroblasts (*p* < 0.0001). Importantly, Tukey's test demonstrated a significant decrease in ROS in both control‐ and XDP‐derived fibroblasts treated with verdiperstat compared with vehicle‐treated control (*p* = 0.052) and XDP fibroblasts (*p* < 0.0001). (C) Two‐way ANOVA demonstrated a significant effect of genotype ([*F* (4, 48) = 91.45], *p* < 0.0001), and time ([*F* (12, 48) = 3.579], *p* = 0.0008) on ROS levels in a second set of fibroblasts. As expected, there was a significant increase in antimycin A‐treated fibroblasts compared with vehicle‐treated control fibroblasts (*p* < 0.0001). Furthermore, Tukey's test demonstrated a significant increase in ROS in XDP fibroblasts compared with control fibroblasts (*p* = 0.0025). Importantly, Tukey's test revealed a significant decrease in ROS levels in both control‐ and XDP‐derived fibroblasts treated with verdiperstat compared with vehicle‐treated control (*p* < 0.0001) and XDP fibroblasts (*p* = 0.0002). **p < 0.001; ****p < 0.0001.

Next, we assessed the effects of a potent and selective MPO inhibitor, verdiperstat, on MPO activity in control‐ and XDP‐derived fibroblasts. The cells were treated with verdiperstat (0.1, 0.5, 1, 5, and 10 μg/mL), for 24 h and MPO activity was measured with a commercially available kit. Verdiperstat treatment significantly decreased MPO activity in a dose‐dependent manner. Specifically, there was a significant decrease in MPO activity in XDP‐derived fibroblasts treated with either 1, 5 or 10 μg/mL of verdiperstat compared to vehicle‐treated XDP‐derived fibroblasts (Figure 5A). Based on these results, we used 5 μg/mL of verdiperstat for the next set of experiments outlined below given that this dose decreased MPO activity by 50%.

Next, the effect of verdiperstat on ROS levels was measured in control‐ and XDP‐derived fibroblasts using CellROX and live cell imaging as described above. The results confirmed a significant increase in ROS in XDP‐derived fibroblasts compared with control‐derived fibroblasts (Figure 5B,C). Importantly, verdiperstat treatment reduced ROS in both control‐ and XDP‐derived fibroblasts (Figure 5B,C).

### 3.6 Verdiperstat Reduced MPO Activity and ROS in XDP‐Treated SH‐SY5Y Cells

**FIGURE 6:**
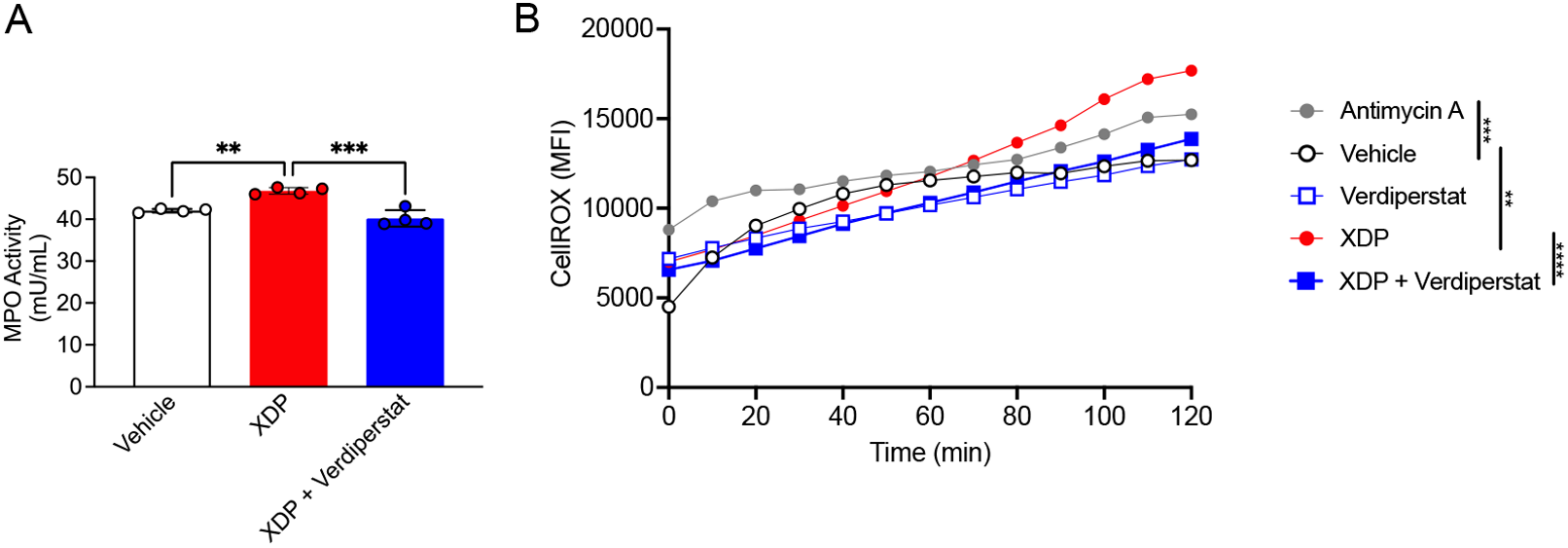
Verdiperstat decreased MPO activity and ROS levels in XDP‐treated SH‐SY5Y cells. (A) One‐way ANOVA revealed a significant effect of treatment ([*F* (2, 9) = 29.19], *p* = 0.0001) on MPO activity in SH‐SY5Y cells. Tukey's test revealed a significant increase in MPO activity in XDP treated cells compared with both vehicle (*p* = 0.0012) and XDP+verdiperstat treated cells (*p* = 0.0001). (B) Two‐way ANOVA revealed a significant effect of treatment ([*F* (4, 48) = 16.35], *p* < 0.0001), and time ([*F* (12, 48) = 31.68], *p* < 0.001) on ROS levels in SH‐SY5Y cells. As expected, ROS levels were increased in antimycin A treated cells compared with vehicle treatment (Tukey's test, *p* = 0.0003). Additionally, Tukey's test demonstrated a significant increase in ROS in XDP treated cells compared with both vehicle (*p* = 0.0014) and XDP+verdiperstat treated cells (*p* < 0.0001, respectively). **p < 0.01; ***p < 0.001; ****p < 0.0001.

To verify whether verdiperstat was also able to reduce MPO activity and ROS in SH‐SY5Y cells treated with postmortem XDP PFC, the cells were treated with XDP PFC homogenates (10 ng/mL) for 24 h in the absence or in the presence of verdipestat (5 μg/mL) and MPO activity was measured as previously described. The results confirmed a significant increase in MPO activity in XDP treated SH‐SY5Y cells compared with vehicle treated cells. Importantly, there was a significant decrease in MPO activity in XDP+verdiperstat treated SH‐SY5Y cells compared with XDP treated SH‐SY5Y cells (Figure 6A).

Lastly, we measured ROS in SH‐SY5Y cells following treatment with XDP (10 ng/mL) for 24 h in the absence or in the presence of verdiperstat (5 μg/mL) using CellROX and live cell imaging. The results demonstrated a significant increase in ROS in SH‐SY5Y cells treated with the positive control antimycin A as well as in XDP treated cells compared with vehicle treated cells. Importantly, there was a significant decrease in ROS in XDP+verdiperstat treated SH‐SY5Y cells compared with XDP treated cells (Figure 6B).

## 4 Conclusion

In this study, we demonstrated a significant increase in MPO activity ex vivo in a large cohort of XDP human postmortem PFC using a novel MPO fluorescent imaging probe [13]. While our results revealed that increases in MPO did not alter pro‐ and anti‐inflammatory cytokine levels, it contributed to a significant increase in ROS in XDP. Both immunodepletion of MPO as well as inhibition of MPO using verdiperstat reduced MPO activity and resulted in significant decreases in ROS in XDP‐derived fibroblasts and SH‐SY5Y cells treated with postmortem XDP PFC. Collectively, these findings suggest that inhibiting MPO dampens oxidative stress in XDP.

Recently, we demonstrated a significant increase in neuroinflammation and MPO in XDP postmortem PFC [14], highlighting MPO as a contributor to this neuroinflammatory process similar to other neurodegenerative diseases. Indeed, increases in MPO levels, astrogliosis and microgliosis have been described in several neurodegenerative diseases, including AD [16, 41, 42], PD [17, 42, 43], and multiple sclerosis (MS) [18, 44, 45].

MPO plays a critical role in the innate immune response as it contributes to both neutrophil antimicrobial activity and phagocytosis [15]; however, a prolonged increase in the systemic levels of MPO could cause extravasation into extracellular spaces, causing tissue damage during chronic inflammation [24, 25]. One consequence of the sustained increase in MPO is the rise in the expression and release of proinflammatory cytokines [21, 38, 39]. However, our findings suggest that increases in MPO levels and activity in XDP, specifically, did not alter cytokine levels. It should be noted that one caveat of the current study is that we only assessed a panel of 10 cytokines and that we did not fully characterize the effects of MPO on a complete panel of inflammatory markers. Therefore, it is possible that increases in MPO may alter the expression and release of other cytokines or chemokines such as CXCL13 and CX3CL1 as we have previously demonstrated in XDP‐derived fibroblasts [14]. Another downstream consequence of increased MPO is oxidative stress and an increase in ROS production through the generation of hypochlorous acid [22, 23]. Here, we report a significant increase in ROS in both XDP‐derived fibroblasts and XDP PFC‐treated SH‐SY5Y cells. Furthermore, the increase in ROS is a direct consequence of increased MPO levels in XDP as it was mitigated by decreasing MPO by either immunodepletion or treatment with verdiperstat. Together these findings demonstrate that inhibiting MPO could represent a viable therapeutic strategy to dampen oxidative stress in XDP, although future studies need to determine whether decreasing ROS could prevent or reduce neurodegeneration and demonstrate clinical benefit.

Verdiperstat is a first‐in‐class, potent, selective, brain‐permeable MPO inhibitor produced by Biohaven Pharmaceuticals Inc. Treatment with verdiperstat has been shown to decrease microglia activation in multiple system atrophy (MSA) [46] and PD [43]. Additionally, increases in MPO levels and activity have been described in human postmortem brain from people who lived with MSA as well as in a transgenic MSA mouse model [46]. Of note, the treatment of MSA mice with verdiperstat reduced MPO activity and ameliorated the disease phenotype [46], thus providing a strong rational for assessing the potential beneficial effect of verdipestat in a clinical trial (NCT03952806 or M‐STAR study). Similarly, increases in MPO were reported in the SOD1^G93A^ animal model of ALS [47, 48], another neurodegenerative disease in which microglia activation plays a critical pathogenic role [49], thus suggesting that verdiperstat could exert a therapeutic effect in ALS and providing the groundwork for assessing verdiperstat in a clinical trial for ALS (NCT04436510 or Regimen B in the Healey ALS Platform Trial in ALS). Although both clinical trials demonstrated safety and tolerability in people, they failed to meet the primary endpoints. Nevertheless, the potential beneficial effect of verdiperstat in treating neurodegenerative diseases is still under investigation and a third clinical trial (Veri‐T) is currently ongoing to determine the therapeutic efficacy of verdiperstat for the treatment of semantic variant primary progressive aphasia (svPPA) (NCT05184569). Therefore, our findings in XDP may pave the way for further assessing the potential beneficial therapeutic effect of verdiperstat in dampening neurodegeneration in XDP.

In summary, our findings demonstrate that increases in MPO may contribute to XDP pathogenesis, as revealed by a significant increase in MPO activity ex vivo in human postmortem XDP PFC. Additionally, our results indicate that MPO causes oxidative stress in XDP, as demonstrated by increases in ROS in cellular models of XDP. Importantly, depleting MPO or using a selective and potent MPO inhibitor mitigated ROS levels in XDP and lays the foundation for future studies to assess the neuroprotective effects of MPO inhibition in XDP.

## Author Contributions

T.P. contributed to conceptualization, data curation, formal analysis, funding acquisition, investigation, methodology, software, supervision, validation, visualization, and writing – original draft. N.J.M., R.Z.B.M., D.L., and M.G.M. contributed to data curation, formal analysis, visualization, and writing – review and editing. E.B.P., D.C.B., C.F.‐C., G.P.L., M.S., E.M., M.C.A., C.C.E.D., C.Z., R.E.T., I.A.Q., and J.W.C. contributed to supervision and writing – review and editing. G.S.‐V. contributed to conceptualization, data curation, funding acquisition, investigation, project administration, resources, supervision, validation, visualization, and writing – original draft.

## Ethics Statement

The study was approved by the Partners Healthcare Institutional Review Board at Massachusetts General Hospital (Boston, MA, USA) and by the Makati Medical Center (Makati City, Philippines).

## Consent

Postmortem consent was obtained from the appropriate representative (next of kin or health care proxy) prior to autopsy.

## Conflicts of Interest

The authors declare no conflicts of interest.

## Supporting information


Figure S1.

## Data Availability

The data supporting the findings of this study are available within the article and/or its Supporting Information.
