# Supplementary figures and images for: Targeting Myeloperoxidase to Reduce Neuroinflammation in X‐Linked Dystonia Parkinsonism
Source: CNS Neurosci Ther. 2024 Nov 5;30(11):e70109. doi: 10.1111/cns.70109 (PMC11537767; doi:10.1111/cns.70109)

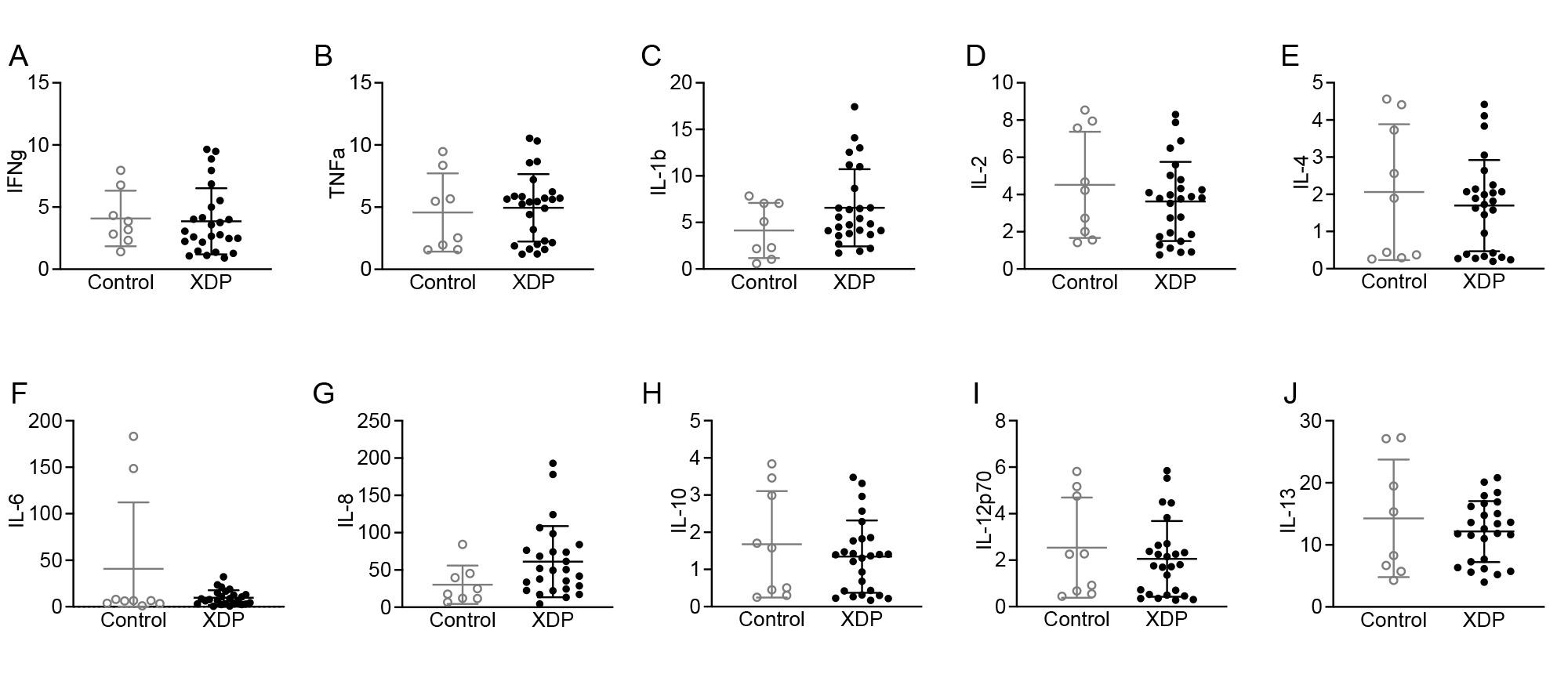

Supplement: Supplementary file 1 — Figure S1. [file CNS-30-e70109-s001.jpg]
